# Ivabradine for coronary artery disease and/or heart failure—a protocol for a systematic review of randomised clinical trials with meta-analysis and Trial Sequential Analysis

**DOI:** 10.1186/s13643-019-0957-0

**Published:** 2019-02-01

**Authors:** M. Maagaard, E. E. Nielsen, C. Gluud, J. C. Jakobsen

**Affiliations:** 1 Copenhagen Trial Unit, Centre for Clinical Intervention Research, Department 7812, Rigshospitalet, Copenhagen University Hospital, Blegdamsvej 9, DK-2100 Copenhagen Ø, Denmark; 2 Cochrane Hepato-Biliary Group, Copenhagen Trial Unit, Centre for Clinical Intervention Research, Department 7812, Rigshospitalet, Copenhagen University Hospital, Copenhagen, Denmark; 3 Department of Cardiology, Holbaek Hospital, Holbaek, Denmark

**Keywords:** Coronary artery disease, Angina pectoris, Heart failure, Ivabradine, Systematic review, Meta-analysis, Trial Sequential Analysis

## Abstract

**Background:**

Coronary artery disease and heart failure are both highly prevalent diseases with a global prevalence of 93 million and 40 million. These patients are at increased risk of morbidity and mortality. The management of these patients involves medical therapy, and both diseases can be treated using the heart rate-lowering drug ivabradine. However, the evidence regarding the use of ivabradine in the treatment of coronary artery disease and/or heart failure is unclear. Our objective is to assess the beneficial and harmful effects of ivabradine in the treatment of coronary artery disease and/or heart failure.

**Methods:**

This protocol for a systematic review was undertaken using the recommendations of The Cochrane Collaboration, the Preferred Reporting Items for Systematic Reviews and Meta-Analysis Protocols (PRISMA-P), and the eight-step assessment procedure suggested by Jakobsen and colleagues. We plan to include all relevant randomised clinical trials assessing the use of ivabradine in the treatment of coronary artery disease and/or heart failure. We will search the Cochrane Central Register of Controlled Trials (CENTRAL), Medical Literature Analysis and Retrieval System Online (MEDLINE), Excerpta Medica database (EMBASE), Latin American and Caribbean Health Sciences Literature (LILACS), Science Citation Index Expanded on Web of Science, Chinese Biomedical Literature Database (CBM), China National Knowledge Infrastructure (CNKI), Chinese Science Journal Database (VIP), and BIOSIS in order to identify relevant trials. We will begin the searches in February 2019. All included trials will be assessed and classified at low risk of bias or at high risk of bias. Our primary conclusions will be based on the results from the primary outcomes at low risk of bias. Extracted data will be analysed using Review Manager 5.3 and Trial Sequential Analysis 0.9.5.10. We will create a ‘Summary of Findings’ table in which we will present our primary and secondary outcomes, and we will assess the quality of evidence using the Grading of Recommendations Assessment, Development and Evaluation (GRADE).

**Discussion:**

The systematic review will have the potential to aid clinicians in decision-making regarding ivabradine and to benefit patients with coronary artery disease and/or heart failure.

**Systematic review registration:**

PROSPERO CRD42018112082

**Electronic supplementary material:**

The online version of this article (10.1186/s13643-019-0957-0) contains supplementary material, which is available to authorized users.

## Description of the conditions

Coronary artery disease is thought to be the most common risk factor and underlying disease of angina pectoris and heart failure [1, 2]. Coronary artery disease can lead to angina pectoris through a decrease in myocardial oxygen supply and to heart failure through acute myocardial infarction, pathological cardiac remodelling, and chronic myocardial dysfunction mediated by myocardial hypoperfusion [3].

### Coronary artery disease and angina pectoris

#### Epidemiology

Cardiovascular diseases account for 30% of all deaths worldwide with ischaemic heart disease (e.g. coronary artery disease) accounting for almost half of all cardiovascular deaths [4, 5]. The prevalence of coronary artery disease increased by 65% between 1990 and 2013, leading to an estimated global prevalence of approximately 93 million in 2013 [6] and a prevalence in the USA of more than 16.5 million [7]. The prevalence is higher in males [7]. Important risk factors for developing coronary artery disease and thereby angina pectoris are smoking, dyslipidaemia, age, hypertension, hyperglycaemia, physical inactivity, obesity, and diet [8–10]. Coronary artery disease is an economic burden with an estimated cost of $ 200 billion in the USA in 2015, whereas the total expenditure for cardiovascular diseases is estimated to be close to $ 550 billion in the USA in 2015 [7].

#### Angina

Coronary artery disease is characterised by recurrent, reproducible episodes of a mismatch between myocardial oxygen supply and myocardial oxygen demand resulting in myocardial ischaemia [11]. The most common manifestation of coronary artery disease is exercise or stress-induced chest discomfort known as stable angina pectoris, and often the diagnosis of angina is based solely on the symptoms as presented by the patient [11–14].

Coronary artery disease is a continuum of clinical presentations most commonly with symptoms of stable angina pectoris or with angina equivalent symptoms (e.g. dyspnoea). The clinical presentation differs due to the underlying pathophysiological mechanisms [11, 15]. There are three mechanisms thought to be the main mediators of angina pectoris: atherosclerotic disease (obstructive coronary artery disease), vasospastic disease (coronary artery spasm), and microvascular dysfunction [16–18]. The three mechanisms all lead to ischaemia, and thereby angina pectoris, by a decreased coronary blood flow due to narrowing of the lumen in the coronary arteries in atherosclerotic and vasospastic disease [16, 18] and in the pre-arterioles and arterioles in microvascular dysfunction [17].

#### Diagnosis and prognosis

The severity of coronary artery disease can be assessed by either of two validated questionnaires—the Rose Questionnaire for Angina Pectoris and the Seattle Angina Questionnaire [19, 20]. Guidelines recommend diagnostic workup to consist of an initial non-invasive test (coronary computed tomography, stress test with exercise electrocardiography, or stress imaging with myocardial perfusion scintigraphy) [11]. The non-invasive tests are not diagnostic but are indicative of coronary artery disease warranting invasive testing [21–23]. However, invasive testing with coronary arteriography should be reserved for patients that are unable to undergo stress testing and where the result will influence patient management (e.g. they are eligible for revascularisation) [11, 24]. In people with coronary artery disease, the annual risk of death appears to be similar to the general population with non-cardiovascular causes being responsible for most fatalities [25]. A review summarising results of non-randomised studies on the prognosis of angina pectoris in primary care estimated the yearly all-cause mortality rate to be 2.8 to 6.6% [26].

### Heart failure

#### Epidemiology

Heart failure is a prevalent disease with an estimated global prevalence of up to 37.7 million people [27, 28]. The lifetime risk for developing heart failure is approximately 20% [29]. The prevalence of heart failure is increasing, presumably caused by an increase in life-expectancy, improved treatment of acute cardiovascular events, and due to risk factors being more prevalent in the populations [27, 28, 30–33]. Risk factors for developing heart failure are hypertension, coronary artery disease, diabetes mellitus, and metabolic syndrome [29]. Heart failure is a costly disease with the USA spending $ 30.8 billion in 2012 and with estimated worldwide costs of $ 108 billion in 2012 [29, 34].

#### Definition and classification

Heart failure is a syndrome characterised by symptoms such as peripheral oedema, fluid retention, breathlessness, and dyspnoea [29]. Heart failure can occur due to functional cardiac disease (e.g. decrease in myocardial function) or structural cardiac disease (e.g. cardiac valve pathology), which may lead to either of or a combination of forward and backward failures [29, 35]. Forward heart failure of the left ventricle is defined as a decrease in cardiac output by means of impaired ejection or filling. Symptoms of forward failure are fatigue, dizziness, confusion, and asthenia (weakness) [36, 37]. Backward failure of the left ventricle is defined as pressure backing up from the left ventricle to the lungs, resulting in increased pulmonary blood pressure and retention of fluid in the lungs leading to dyspnoea and pulmonary oedema. Right ventricular failure might occur due to left ventricular failure, increased pulmonary blood pressure, or myocardial infarction. The symptoms of right ventricular failure are hepatomegaly, elevated jugular venous pressure, and peripheral oedema [38]. Decompensated (congestive) heart failure is the clinical syndrome of heart failure in relation to fluid and salt retention [29].

Heart failure can be classified according to left ventricular ejection fraction and is grouped into heart failure with preserved ejection fraction (ejection fraction ≥ 50%), heart failure with borderline/mid-range ejection fraction (ejection fraction 40 to 49%), and heart failure with reduced ejection fraction (ejection fraction < 40%) [29, 35]. The method most commonly used in staging heart failure is the New York Heart Association (NYHA) classification that stages heart failure according to the physical capabilities of an individual with heart failure [29, 35, 39].

#### Pathophysiology

Heart failure is the end stage of several diseases affecting the heart [40]. Heart failure is initiated by an index event that happens either abruptly (e.g. acute myocardial infarction) or gradually (e.g. genetic cardiomyopathies, ischaemic heart disease, or valve pathology) [41]. Following the index event, compensatory mechanisms attempt to maintain haemodynamic stability by means of maintaining an adequate cardiac output [41]. The main compensatory mechanism is the activation of the sympathetic nervous system led by the renin-angiotensin-aldosterone system [41, 42]. Prolonged activation of compensatory mechanisms might lead to the development of cardiac fibrosis, endothelial dysfunction, atherosclerosis, cardiomyopathy, cardiac remodelling, left ventricular hypertrophy, and aortic stiffening [42]. In pathological cardiac remodelling, the ventricles alter their function, shape, and size leading to increased afterload, subendocardial hypoperfusion, increased myocardial oxygen demand, and myocardial desynchronization [40, 43].

#### Diagnosis and prognosis

The diagnosis of heart failure is based on a thorough clinical examination of patients [29]. Paraclinical tests should include, but are not limited to, a 12-lead electrocardiogram and blood sampling [29]. Echocardiography is a low-cost, easy to apply modality for assessing the structure (e.g. size of chambers) and function of the heart (e.g. valve function and systolic and diastolic function). Traditionally, left ventricular ejection fraction measured by transthoracic echocardiography is the mainstay in the diagnosis and management of heart failure [44]. An observational study on the survival following a hospitalisation for incident heart failure found the all-cause mortality at 1, 2, and 5 years to be 37.3%, 52.9%, and 78.5% [45]. From the Framingham Heart Study, the 30-day mortality was 11% in men and 10% in women and the 5-year mortality was 59% in men and 45% in women [46]. Studies on the temporal trends of mortality related to heart failure have shown a decrease in mortality over time [46, 47]. The decrease in mortality is thought to be due to improved treatment of acute and chronic heart failure, early diagnosis, and partly due to a decrease in the prevalence of risk factors such as smoking [28].

## Description of the intervention

### Treatment of coronary artery disease

The purpose of the treatment of coronary artery disease is well established across different guidelines: prevent premature death, prevent acute myocardial infarction and heart failure, minimise or eliminate symptoms of ischaemia, and restore or maintain physical activity and life quality [11, 24]. The treatment of coronary artery disease is focused on preventing attacks of angina and treating attacks when they occur [48]. These ‘anti-anginal drugs’ exert their effect in preventing and treating angina pectoris by decreasing myocardial oxygen consumption by means of decreasing heart rate, blood pressure, and myocardial contractility or by increasing the myocardial oxygen supply, mainly by means of increasing the coronary blood flow [48]. The 2013 European Society of Cardiology and the 2012 American College of Cardiology/American Heart Association guidelines recommend the following interventions for preventing and treating angina pectoris in coronary artery disease:

First line of treatment: beta-blocker or calcium channel blocker alone or in combination [11, 24]

Second line of treatment: switching to or adding either long-acting nitrates, ivabradine, nicorandil, ranolazine, or trimetazidine [11, 24]

### Treatment of heart failure

The purpose of the treatment of heart failure is to improve clinical status, functional capabilities, and quality of life; prevent hospitalisation; and reduce mortality [29, 35]. Guidelines recommend the main treatment of heart failure with reduced ejection fraction to be a beta-blocker in addition to an angiotensin-converting enzyme inhibitor. For patients with heart failure with reduced ejection fraction who remain symptomatic despite treatment with an angiotensin-converting enzyme inhibitor and a beta-blocker, the addition of a mineralocorticoid-receptor antagonist is recommended [29, 35]. In addition, diuretics are used in patients with volume overload and in patients with decompensated heart failure as well as being the main component in the treatment of patients with heart failure with preserved ejection fraction [29, 35].

### Ivabradine

Ivabradine is used in the treatment of coronary artery disease and heart failure [11, 29, 35, 49]. Ivabradine is administered in tablet form from 2.5 to 7.5 mg twice daily in both coronary artery disease and heart failure [11, 35].

In the normal heart, the sinoatrial node is the dominant site of pacemaker activity through the I_f_-channel that spontaneously generates action potentials when the cell becomes hyperpolarised. Thereby, the sinoatrial node is responsible for the heart rate [50]. Ivabradine selectively inhibits the I_f_-channel, resulting in a decrease in action potentials and thereby a decrease in heart rate [50]. Ivabradine selectively decreases the heart rate, but unlike beta-blockers, ivabradine does not inhibit the inner conduction system of the heart [50]. Unlike beta-blockers and heart rate-lowering calcium channel antagonists, ivabradine does not exert a negative inotropic effect (the force of muscular contraction of the heart) [50].

Ivabradine is recommended in the European Society of Cardiology guidelines on acute and chronic heart failure for stable, symptomatic patients with left ventricular ejection fraction below or equal to 35% in sinus rhythm with a resting heart rate above or equal to 70 beats per minute despite therapy with beta-blocker, angiotensin-converting enzyme inhibitor (or angiotensin receptor blocker), or a mineralocorticoid receptor antagonist, or for people in whom beta-blocker therapy is contraindicated [35]. Ivabradine is also recommended as the second-line therapy for angina relief in the European Society of Cardiology guidelines on stable coronary artery disease [11]. Ivabradine was not included as a recommendation in the 2012 American College of Cardiology Foundation/American Heart Association guidelines on ischaemic heart disease, since the drug was first approved by the Food and Drug Administration in the USA in 2015 [24, 51].

In both heart failure and coronary artery disease, ivabradine might be beneficial due to its ability to selectively decrease heart rate, thereby decreasing myocardial oxygen demand. Theoretically, ivabradine also increases myocardial oxygen supply by prolonging diastole, thereby increasing the coronary artery perfusion without inhibiting the force of muscular contraction of the heart [50, 52, 53].

CYP3A4 inhibitors (e.g. clarithromycin, atazanavir, ketoconazole) may increase the plasma concentration of ivabradine, and adverse events associated with ivabradine are bradycardia, hypertension, atrial fibrillation, and phosphenes [54].

## Why is it important to do this review?

Several reviews of randomised clinical trials have assessed the effect of ivabradine [55–58]. A review of randomised trials on ivabradine in the treatment of systolic heart failure found that treatment with ivabradine did not improve isolated cardiovascular death, isolated heart failure hospitalisation, or all-cause mortality [55]. A recent review of randomised trials on ivabradine for heart failure or coronary artery disease found that mortality in ivabradine-treated groups might not differ from groups treated with placebo [56]. Another review of randomised trials on the effectiveness of ivabradine in treating stable angina pectoris found that ivabradine significantly increased exercise duration and time to onset of angina [57]. Yet another review of randomised trials on ivabradine for coronary artery disease with or without left ventricular dysfunction found that ivabradine did not seem to affect the risk of all-cause mortality, cardiovascular death, or hospitalisation for new onset or worsening heart failure [58]. A review on ivabradine for stable angina also found that ivabradine does not seem to affect the risk of all-cause mortality, cardiovascular mortality and morbidity, or hospitalisation for worsening heart failure [59]. However, none of the above-mentioned reviews were systematic reviews, none adhered to the Preferred Reporting Items for Systematic reviews and Meta-analyses (PRISMA) guidelines, none of them published a protocol, none of them used Trial Sequential Analysis to minimise the risk of random errors, and only the 2016 review by Ye et al. and the 2017 review by Mengesha et al. assessed the risk of bias in individual trials according to The Cochrane Collaboration risk of bias in individual trial tool [57, 60–65]. There is a need for an updated systematic review assessing the harms and benefits of ivabradine for coronary artery disease and/or heart failure, adhering to the PRISMA statement, searching all relevant databases, assessing risk of bias in individual trials, minimising the risk of random error by means of Trial Sequential Analysis, and assessing the certainty of the evidence with GRADE [60, 61, 66–68].

The question sought to be answered is:

In patients with coronary artery disease and/or heart failure, does ivabradine compared with placebo benefit any clinical outcomes or induce any harms?

## Methods

This protocol for a systematic review has been developed based on the Preferred Reporting Items for Systematic Reviews and Meta-Analysis Protocols (PRISMA-P) guidelines for reporting systematic reviews evaluating interventions in healthcare [66, 69] and the Cochrane Handbook [60].

### Criteria for considering studies for this review

#### Types of studies

We will include randomised clinical trials irrespective of trial design, setting, publication status, publication year, and language for assessment of benefits and harms. We will include cluster-randomised studies. We will not include quasi-randomised studies or observational studies for the assessments of benefits. However, such non-randomised studies identified during our searches for trials will be included in the review for the assessments of adverse events. We are aware that by not searching for all observational studies on harms, we run the risk of putting more weight on potential benefits than on potential harms and we are likely to overlook rare adverse events and late adverse events [70].

#### Types of participants

We will include participants with coronary artery disease and/or heart failure. We will include participants irrespective of age, sex, and comorbidities.

#### Types of interventions

Experiment intervention: ivabradine in any form or dose

Control intervention: placebo or no intervention

We will accept any co-intervention, if the co-intervention is intended to be delivered similarly to the intervention and control groups.

#### Types of outcomes

For all outcomes, we will use the trial results reported at maximum follow-up. However, if the trialists report results at multiple time points, we will use the results reported at maximum follow-up.

#### Primary outcomes

1. All-cause mortality
2. Proportion of participants with one or more serious adverse events. We will define a serious adverse event as any untoward medical occurrence that resulted in death, was life-threatening, required hospitalisation or prolongation of existing hospitalisation, and resulted in persistent or significant disability or jeopardised the patient [71] (dichotomous outcome)
3. Quality of life measured on any valid scale (continuous outcome)

#### Secondary outcomes

1. Cardiovascular mortality
2. Myocardial infarction (dichotomous outcome)
3. Proportion of participants with one or more adverse events not considered serious (please see above)

#### Exploratory outcomes

- Resting heart rate (continuous outcome)
- Ejection fraction (continuous outcome)
- Six-minute walking distance (continuous outcome)
- Angina measured on any valid scale (continuous outcome)
- New York Heart Association (NYHA) classification of heart failure (dichotomous outcome)
- Hospitalisation during follow-up (dichotomous outcome)
- Individual serious adverse events
- Individual adverse events not considered serious

### Search methods

#### Electronic searches

We will search the Cochrane Central Register of Controlled Trials (CENTRAL), Medical Literature Analysis and Retrieval System Online (MEDLINE), Excerpta Medica database (EMBASE), Latin American and Caribbean Health Sciences Literature (LILACS), Science Citation Index Expanded on Web of Science, Chinese Biomedical Literature Database (CBM), China National Knowledge Infrastructure (CNKI), Chinese Science Journal Database (VIP), and BIOSIS in order to identify relevant trials. The preliminary search strategy for MEDLINE (Ovid) is given in Additional file 1. We will search all databases from their inception to the present. We will begin the search in February 2019.

#### Searching other resources

The reference lists of relevant publications will be checked for any unidentified randomised trials. We will contact authors of included studies and major pharmaceutical companies involved in the production or sales of ivabradine by email asking for unpublished randomised trials. Further, we will search for ongoing trials on:

- ClinicalTrials.gov (www.clinicaltrials.gov)
- Google Scholar (https://scholar.google.dk/)
- The Turning Research into Practice (TRIP) Database (https://www.tripdatabase.com)
- European Medicines Agency (EMA) (https://www.ema.europa.eu/ema/)
- US Food and Drug Administration (FDA) (www.fda.gov)
- China Food and Drug Administration (CFDA) (http://eng.sfda.gov.cn/WS03/CL0755/)
- Medicines and Healthcare products Regulatory Agency (https://www.gov.uk/government/organisations/medicines-and-healthcare-products-regulatory-agency)
- The World Health Organization (WHO) International Clinical trials Registry Platform (ICTRP) search portal (http://apps.who.int/trialsearch)

Additionally, we will by hand search conference abstracts from cardiology conferences for relevant trials. We will also consider unpublished and grey literature trials relevant to the review, if we identify such trials.

### Data collection and analysis

We will perform the review following the recommendations of The Cochrane Collaboration [60]. The analyses will be performed using Review Manager [72] and Trial Sequential Analysis [73]. In case of Review Manager statistical software not being sufficient, we will use STATA 15 [74].

#### Selection of studies

Two review authors (MM and EEN) will independently screen titles and abstracts. We will retrieve all relevant full-text study reports and publications. Two review authors (MM and EE) will independently screen the full text and identify and record reasons for exclusion of the ineligible studies. We will resolve any disagreement through discussion or, if required, we will consult a third author (JCJ). Trial selection will be displayed in an adapted flow diagram as per the Preferred Reporting Items for Systematic Reviews and Meta-Analyses (PRISMA) statement [61].

#### Data extraction and management

Two authors (MM and EEN) will extract data independently from included trials. Disagreements will be resolved by discussion with a third author (JCJ). We will assess duplicate publications and companion papers of a trial together to evaluate all available data simultaneously (maximise data extraction, correct bias assessment). We will contact the trial authors by email to specify any additional data, which may not have been reported sufficiently or at all in the publication.

#### Trial characteristics

Bias risk components (as defined below), trial design (parallel, cluster, factorial, or crossover), number of intervention arms, length of follow-up, estimation of sample size, and inclusion and exclusion criteria.

#### Participant characteristics and diagnosis

Number of randomised participants; number of analysed participants; number of participants lost to follow-up/withdrawals/crossover; compliance with medication; age range (mean or median) and sex ratio; type of arrhythmia (atrial fibrillation or atrial flutter); baseline numbers of cardiovascular risk factors (i.e. diabetes mellitus, hypertension, hyperlipidaemia, or smoking); baseline NYHA class; baseline number of participants with heart failure; baseline number of participants with valvular heart disease; baseline number of participants with previous myocardial infarction; baseline number of participants with previous revascularisation; and baseline number of participants with previous angina. We will additionally report the proportion of participants in the compared groups who receive beta-blockers, calcium channel blockers, long or short acting nitrates, angiotensin converting enzyme inhibitors, angiotensin II-receptor antagonists, angiotensin II-neprilysin inhibitors, and/or mineralocorticoid receptor antagonists.

#### Ivabradine strategy characteristics

Dose of intervention, mode of administration, and duration of administration

#### Co-intervention characteristics

Type of co-intervention, dose of co-intervention, duration of co-intervention, and mode of administration

#### Individual patient data

We will ask those responsible for the included trials to supply individual patient data.

#### Notes

Funding of the trial and notable conflicts of interest of trial authors will be extracted, if available. We will note in the ‘Characteristics of included studies’ table if outcome data were not reported in a usable way. Two review authors (MM and EEN) will independently transfer data into the Review Manager file. Disagreements will be resolved through discussion, or if required, we will consult with a third author (JCJ).

### Assessment of risk of bias in included studies

We will use the instructions given in the Cochrane Handbook for Systematic Reviews of Interventions in our evaluation of the methodology and hence the risk of bias of the included trials. We will evaluate the methodology in respect of:

- Random sequence generation
- Allocation concealment
- Blinding of participants and treatment providers
- Blinding of outcome assessment
- Incomplete outcome data
- Selective outcome reporting
- For profit bias
- Other risks of bias
- Overall risk of bias

These components enable classification of randomised trials with low risk of bias and high risk of bias. The latter trials tend to overestimate positive intervention effects and underestimate negative effects [75–81]. We will classify a trial as being at overall ‘low risk of bias’, only if all bias domains are classified as ‘low risk of bias’. We will classify a trial as being at overall ‘high risk of bias’, if any of the bias domains are classified as ‘unclear’ or ‘high risk of bias’. We will also assess for profit bias (see the ‘Appendix’ section for a list of criteria that we will classify the trials according to).

We will assess the domains ‘blinding of outcome assessment’, ‘incomplete outcome data’, and ‘selective outcome reporting’ for each outcome result. Thus, we can assess the bias risk for each outcome assessed in addition to each trial. Our primary conclusions will be based on the results of our primary outcomes at overall low risk of bias.

#### Differences between the protocol and the review

We will conduct the review according to this published protocol and report any deviations from it in the ‘Differences between the protocol and the review’ section of the systematic review.

### Measures of treatment effect

#### Dichotomous outcomes

We will calculate risk ratios (RRs) with 95% confidence interval (CI) for dichotomous outcomes, as well as the Trial Sequential Analysis-adjusted CIs (see below).

#### Continuous outcomes

We will calculate the mean differences (MDs) with 95% CI for continuous outcomes, as well as the Trial Sequential Analysis-adjusted CIs (see below).

### Dealing with missing data

We will, as first option, contact all trial authors to obtain any relevant missing data (i.e. for data extraction and for assessment of risk of bias, as specified above).

#### Dichotomous outcomes

We will not impute missing values for any outcomes in our primary analysis. In two of our sensitivity analyses (see paragraph below), we will impute data.

#### Continuous outcomes

We will primarily analyse scores assessed at single time points. If only changes from baseline scores are reported, we will analyse the results together with follow-up scores. If standard deviations (SDs) are not reported, we will calculate the SDs using trial data, if possible. We will not use intention-to-treat data if the original report did not contain such data. We will not impute missing values for any outcomes in our primary analysis. In our sensitivity analysis (see paragraph below) for continuous outcomes, we will impute data.

### Assessment of heterogeneity

We will primarily investigate forest plots to visually assess any sign of heterogeneity. We will secondly assess the presence of statistical heterogeneity by chi-square test (threshold *P* < 0.10) and measure the quantities of heterogeneity by the *I*^2^ statistics [82, 83].

We will investigate possible heterogeneity through subgroup analyses. Ultimately, we may decide that a meta-analysis should be avoided [60].

#### Assessment of reporting biases

We will use a funnel plot to assess reporting bias if ten or more trials are included. We will visually inspect funnel plots to assess the risk of bias. We are aware of the limitations of a funnel plot (i.e. a funnel plot assesses bias due to small sample size). From this information, we assess possible reporting bias. For dichotomous outcomes, we will test asymmetry with the Harbord test [84] if *τ*^2^ is less than 0.1 and with the Rücker test if *τ*^2^ is greater than 0.1. For continuous outcomes, we will use the regression asymmetry test [85] and the adjusted rank correlation test [86].

### Unit of analysis issues

We will only include randomised clinical trials. For trials using crossover design, only data from the first period will be included [60, 87]. We will also include cluster-randomised trials after adjusting the original sample size of the trial to the effective sample size using the intracluster correlation coefficient from the ‘design effect’ [60]. Therefore, there will not be any unit of analysis issues.

### Data synthesis

#### Meta-analysis

We will undertake this meta-analysis according to the recommendations stated in the Cochrane Handbook for Systematic Reviews of Interventions [60], Keus et al. [88], and the eight-step assessment suggested by Jakobsen et al. [89]. We will use the statistical software Review Manager 5.3 provided by Cochrane to analyse data [72]. We will assess our intervention effects with both random effects meta-analyses [90] and fixed effects meta-analyses [91]. We will use the more conservative point estimate of the two [89]. The more conservative point estimate is the estimate closest to zero effect. If the two estimates are similar, we will use the estimate with the highest *P* value. We use three primary outcomes, and therefore, we will consider a *P* value of 0.025 as the threshold for statistical significance [89]. For secondary and exploratory outcomes, we will consider a *P* value of 0.05 as the threshold for statistical significance. We will investigate possible heterogeneity through subgroup analyses. Ultimately, we may decide that a meta-analysis should be avoided because of unexpected high heterogeneity [60]. We will use the eight-step procedure to assess if the thresholds for significance are crossed [89]. Our primary conclusion will be based on the results from the primary outcomes at low risk of bias [89]. Where multiple trial groups are reported in a single trial, we will include only the relevant groups. If two comparisons are combined in the same meta-analysis, we will have the control group to avoid double-counting [60]. Trials with a factorial design will be included.

#### Trial Sequential Analysis

Traditional meta-analysis runs the risk of random errors due to sparse data and repetitive testing of accumulating data when updating reviews. We wish to control the risks of type I errors and type II errors. We will, therefore, perform Trial Sequential Analysis on the outcomes, in order to calculate the required information size (that is the number of participants needed in a meta-analysis to detect or reject a certain intervention effect) and the cumulative *Z*-curve’s breach of relevant trial sequential monitoring boundaries [62–65, 73, 92–96]. A more detailed description of Trial Sequential Analysis can be found in the Trial Sequential Analysis manual and at http://www.ctu.dk/tsa/ [94]. For dichotomous outcomes, we will estimate the required information size based on the observed proportion of patients with an outcome in the control group (the cumulative proportion of patients with an event in the control groups relative to all patients in the control groups), a relative risk reduction of 15%, an alpha of 2.5% for primary outcomes, an alpha of 5% for secondary and exploratory outcomes, a beta of 10%, and diversity as suggested by the trials in the meta-analysis. For continuous outcomes, we will in the Trial Sequential Analysis use the observed SD, a mean difference of the observed SD/2, an alpha of 2.5% for primary outcomes, an alpha of 5% for secondary and exploratory outcomes, and a beta of 10%.

### Subgroup analysis and investigation of heterogeneity

#### Subgroup analysis

We will perform the following subgroup analysis when analysing the primary outcomes (all-cause mortality, serious adverse event, and quality of life):

1. Trials at high risk of bias compared to trials at low risk of bias
2. Angina pectoris trials compared to heart failure trials
3. Men compared to women
4. Participants with a resting heart rate at or above 70 compared to participants with a resting heart rate below 70.
5. Trials administering at or above median daily doses of ivabradine compared to trials administering ivabradine below median daily doses.
6. Trials administering at or above median duration (days) of ivabradine compared to trials administering ivabradine below median duration.

We will use the formal test for subgroup interactions in Review Manager [97].

#### Sensitivity analysis

To assess the potential impact of the missing data for dichotomous outcomes, we will perform the two following sensitivity analyses on both the primary and secondary outcomes:

- ‘Best-worst-case’ scenario: we will assume that all participants lost to follow-up in the ivabradine group have survived, had no serious adverse events, had a higher quality of life (see paragraph below), and had no myocardial infarction. We will assume the opposite for all participants lost to follow-up in the control group.
- ‘Worst-base-case’ scenario: we will assume that all participants lost to follow-up in the ivabradine group have not survived, had serious adverse events, had a lower quality of life (see paragraph below), and had myocardial infarction. We will assume the opposite for all participants lost to follow-up in the control group.

We will present results of both scenarios in our review. When analysing quality of life or ejection fraction, a ‘beneficial outcome’ will be the group mean plus two standard deviations (SDs) of the group mean and a ‘harmful outcome’ will be the group mean minus two SDs of the group mean [89].

We will present results of this scenario in our review. Other post hoc sensitivity analyses might be warranted if unexpected clinical or statistical heterogeneity is identified during the analysis of the review results [89].

### ‘Summary of Findings’ table

We will create a ‘Summary of Findings’ table using each of the prespecified outcomes (all-cause mortality, serious adverse event, quality of life, cardiovascular death, myocardial infarction, adverse events not considered to be serious). We will use the five GRADE considerations (bias risk of the trials, consistency of effect, imprecision, indirectness, and publication bias) to assess the quality of a body of evidence as it relates to the studies which contribute data to the meta-analyses for the prespecified outcomes [89, 98–100]. We will use methods and recommendations described in Chapter 8 (Section 8.5) and Chapter 12 of the Cochrane Handbook for Systematic Reviews of Interventions using GRADEpro software [60]. We will justify all decisions to downgrade the quality of studies using footnotes, and we will make comments to aid the reader’s understanding of the review where necessary. First, we will present our results in the ‘Summary of Findings’ table based on the results from the trials with low risk of bias. Secondly, we will present the results based on all trials.

We will compare our GRADE assessment of imprecision based on a fixed effects model calculation of the optimal information size with that obtained with Trial Sequential Analysis based on a random effects model and heterogeneity correction of the required information size [101].

## Discussion

This systematic review protocol has several strengths. We have based the protocol on the Preferred Reporting Items for Systematic Reviews and Meta-Analysis Protocols (PRISMA-P) checklist [66, 69]. We have pre-defined our methodology based on the Cochrane Handbook for Systematic Reviews of Interventions [60], Keus et al. [88], the eight-step assessment as suggested by Jakobsen et al. [89], Trial Sequential Analysis [73], and the GRADE assessment [99, 102]. Through our pre-defined methodology, we consider the risk of random errors and systematic errors.

The systematic review will also have limitations. We will pool data from all trials regarding the treatment of coronary artery disease and heart failure using ivabradine, thereby potentially giving rise to clinical and statistical heterogeneity. However, we believe that there is significant overlap between patients with coronary artery disease and heart failure and the effects of ivabradine might therefore be similar in these different patient groups. Furthermore, we will estimate statistical heterogeneity and ultimately decide if a meta-analysis of all trials should be avoided. Moreover, we have pre-defined several subgroup and sensitivity analysis in order to assess whether or not the intervention effect differs between conditions and trials. We may conduct further subgroup analyses and sensitivity analyses to explain unexplained heterogeneity. By not searching for all non-randomised studies, we will likely overlook harms [70]. If the present review finds solid evidence for benefits, then a more thorough investigation of potential harms seems warranted. With this systematic review, we seek to provide the clinicians and decision-makers on clinical practice with a reliable evidence adjusted for bias, sparse data, and multiple testing regarding the treatment of coronary artery disease and heart failure using ivabradine.

### Additional file

Additional file 1 Preliminary search strategy for MEDLINE (OvidSP; 1946 to October 2018) (DOCX 14 kb)

## Appendix

### Detailed explanation of each bias domain

Random sequence generation

- Low risk: If sequence generation was achieved using computer random number generator or a random number table. Drawing lots, tossing a coin, shuffling cards, and throwing dice were also considered adequate if performed by an independent adjudicator.
- Unclear risk: If the method of randomisation was not specified, but the trial was still presented as being randomised.
- High risk: If the allocation sequence is not randomised or only quasi-randomised. These trials will be excluded.

Allocation concealment

- Low risk: If the allocation of patients was performed by a central independent unit, on-site locked computer, identical-looking numbered sealed envelopes, drug bottles, or containers prepared by an independent pharmacist or investigator.
- Uncertain risk: If the trial was classified as randomised but the allocation concealment process was not described.
- High risk: If the allocation sequence was familiar to the investigators who assigned participants.

Blinding of participants and treatment providers

- Low risk: If the participants and the treatment providers were blinded to intervention allocation and this was described.
- Uncertain risk: If the procedure of blinding was insufficiently described.
- High risk: If blinding of participants and the treatment providers was not performed.

Blinding of outcome assessment

- Low risk of bias: If it was mentioned that outcome assessors were blinded, and this was described.
- Uncertain risk of bias: If it was not mentioned if the outcome assessors in the trial were blinded or the extent of blinding was insufficiently described.
- High risk of bias: If no blinding or incomplete blinding of outcome assessors was performed.

Incomplete outcome data

- Low risk of bias: If missing data were unlikely to make treatment effects depart from plausible values. This could be either (1) there were no drop-outs or withdrawals for all outcomes or (2) the numbers and reasons for the withdrawals and drop-outs for all outcomes were clearly stated and could be described as being similar to both groups. Generally, the trial is judged as at a low risk of bias due to incomplete outcome data if drop-outs are less than 5%. However, the 5% cut-off is not definitive.
- Uncertain risk of bias: If there was insufficient information to assess whether missing data were likely to induce bias on the results.
- High risk of bias: If the results were likely to be biased due to missing data either because the pattern of drop-outs could be described as being different in the two intervention groups or the trial used improper methods in dealing with the missing data (e.g. last observation carried forward).

Selective outcome reporting

- Low risk of bias: If a protocol was published before or at the time the trial was begun, and the outcomes specified in the protocol were reported on. If there is no protocol or the protocol was published after the trial has begun, reporting of all-cause mortality and all serious adverse events will grant the trial a grade of low risk of bias.
- Uncertain risk of bias: If no protocol was published and the outcome all-cause mortality and serious adverse events were not reported on.
- High risk of bias: If the outcomes in the protocol were not reported on.

For profit bias

- Low risk of bias: If the trial is not financed by a company that might have an interest in a given result.
- Uncertain risk of bias: If there is no description of how the trial is financed.
- High risk of bias: If the trial is financed by a company that might have an interest in a given result.

Other risks of bias

- Low risk of bias: If the trial appears to be free of other components (for example, academic bias or for profit bias) that could put it at risk of bias.
- Unclear risk of bias: If the trial may or may not be free of other components that could put it at risk of bias.
- High risk of bias: If there are other factors in the trial that could put it at risk of bias (for example, authors conducted trials on the same topic and for profit bias).

Overall risk of bias

- Low risk of bias: The trial will be classified as overall ‘low risk of bias’ only if all of the bias domains described in the above paragraphs are classified as ‘low risk of bias’.
- High risk of bias: The trial will be classified as ‘high risk of bias’ if any of the bias risk domains described in the above are classified as ‘unclear’ or ‘high risk of bias’.
