# Supplementary material for: Ivabradine for coronary artery disease and/or heart failure—a protocol for a systematic review of randomised clinical trials with meta-analysis and Trial Sequential Analysis
Source: Syst Rev. 2019 Feb 1;8:39. doi: 10.1186/s13643-019-0957-0 (PMC6357471; doi:10.1186/s13643-019-0957-0)
Supplement: Supplementary file 1 — Preliminary search strategy for MEDLINE (OvidSP; 1946 to October 2018) (DOCX 14 kb) [file 13643_2019_957_MOESM1_ESM.docx]

### Additional file 1 Preliminary search strategy for Medline (OvidSP; 1946 to October 2018)

1. exp heart failure/
2. exp coronary disease/
3. heart failure.mp. [mp=title, abstract, original title, name of substance word, subject heading word, floating sub-heading heading word, keyword heading word, protocol supplementary concept word, rare disease supplementary concept word, unique identifier, synonyms]
4. coronary diseases.mp. [mp=title, abstract, original title, name of substance word, subject heading word, floating sub-heading heading word, keyword heading word, protocol supplementary concept word, rare disease supplementary concept word, unique identifier, synonyms]
5. 1 or 2 or 3 or 4
6. ivabradine.mp. [mp=title, abstract, original title, name of substance word, subject heading word, floating sub-heading heading word, keyword heading word, protocol supplementary concept word, rare disease supplementary concept word, unique identifier, synonyms]
7. corlanor.mp. [mp=title, abstract, original title, name of substance word, subject heading word, floating sub-heading heading word, keyword heading word, protocol supplementary concept word, rare disease supplementary concept word, unique identifier, synonyms]
8. procoralan.mp. [mp=title, abstract, original title, name of substance word, subject heading word, floating sub-heading heading word, keyword heading word, protocol supplementary concept word, rare disease supplementary concept word, unique identifier, synonyms]
9. 6 or 7 or 8
10. 5 and 9
11. random*.mp. [mp=title, abstract, original title, name of substance word, subject heading word, floating sub-heading heading word, keyword heading word, protocol supplementary concept word, rare disease supplementary concept word, unique identifier, synonyms]
12. blind*.mp. [mp=title, abstract, original title, name of substance word, subject heading word, floating sub-heading heading word, keyword heading word, protocol supplementary concept word, rare disease supplementary concept word, unique identifier, synonyms]
13. placebo*.mp. [mp=title, abstract, original title, name of substance word, subject heading word, floating sub-heading heading word, keyword heading word, protocol supplementary concept word, rare disease supplementary concept word, unique identifier, synonyms]
14. meta-analys*.mp. [mp=title, abstract, original title, name of substance word, subject heading word, floating sub-heading heading word, keyword heading word, protocol supplementary concept word, rare disease supplementary concept word, unique identifier, synonyms]
15. 11 or 12 or 13 or 14
16. 10 and 15
